# The Effect of Task Difficulty and Self-Contribution on Fairness Consideration: An Event-Related Potential Study

**DOI:** 10.3389/fpsyg.2022.709310

**Published:** 2022-03-03

**Authors:** Liyan Xu, Biye Wang, Wei Guo

**Affiliations:** ^1^College of Physical Education, Yangzhou University, Yangzhou, China; ^2^Institute of Sports, Exercise and Brain, Yangzhou University, Yangzhou, China

**Keywords:** self-contribution, task difficulty, fairness consideration, feedback-related negativity, P300

## Abstract

Self-contribution may be an influential factor in fairness consideration and consequent behavioral decisions. Few studies have investigated simultaneous effects of task difficulty and self-contribution on fairness consideration outcomes and associated neurophysiological responses. To elucidate modulation effects of task difficulty and self-contribution on fairness consideration, 30 recruited participants played a modified ultimatum game (UG) while undergoing event-related potential measurements. A 2 (task difficulty: hard vs. easy) × 3 (contribution: other-contribution vs. both-contribution vs. self-contribution) × 2 (fairness type: fair vs. unfair) within-subject design was adopted. A significant interaction between fairness type and contribution was observed in the behavioral data, with unfair offers being more acceptable in the other-contribution condition than in the self-contribution or both-contribution conditions. In the early processing time window, feedback-related negative magnitudes were greater in the hard condition than in the easy condition. P300 responses were more pronounced when participants contributed equally to the proposer than in the self- and other-contribution conditions. These results demonstrated that individuals’ decisions are influenced by their own effort contributions relative to those of others in cooperative contexts.

## Introduction

Typically, people show strong motivation for sustaining fairness in their interpersonal interactions. When confronted with unfairness, fairness consideration may lead people to sacrifice some financial benefit to themselves to punish others (Fehr and Gachter, 2002; Hu et al., 2014; Fortin et al., 2020; Nai et al., 2020). Although this punitive behavior may not seem beneficial, from an evolutionary perspective, this pattern of behavior can benefit interpersonal interactions in the long term while reducing the probability of similar harm in the future when faced with the same situation (Barclay, 2006; Krasnow and Delton, 2016).

Fairness consideration does not depend solely on direct trade-offs between oneself and others. Contextual factors, such as social distance (Yu et al., 2015; Li et al., 2020), facial attractiveness (Wu et al., 2011; Ma et al., 2017), other’s intentions (Sutter, 2007; Guroglu et al., 2011), self-contribution (Guo et al., 2014; Bland et al., 2017), and task difficulty can influence behavior related to fairness consideration. Among these contextual factors, each individual’s contribution is particularly important. A low-contributing individual may become less likely to be selected as a cooperator. In various real-world settings, allocation based on individual contribution is considered a common and fair approach. The natural desert theory predicts that people expect that their compensation should be based on their own efforts and contributions to collective resources (Hoffman and Spitzer, 1985; Barker et al., 2012). Indeed, in neurophysiological studies, when participants played a more important part in earning activities, they were more likely to reject unfair offers; and greater self-contribution was associated with greater activity in the anterior insula, anterior cingulate cortex, dorsolateral prefrontal cortex, and temporoparietal junction when (Guo et al., 2014; Feng et al., 2015, 2019).

Task difficulty is a somewhat controversial putative factor in fairness consideration. Response time and error rate are the most common indices of task difficulty (Gilbert et al., 2012). For example, by manipulating response time, Wang (2017) found that participants were more likely to make fair decisions in proportion to their contribution when working in an easy condition (neat arrangement of experimental materials) than when working in a hard condition (cluttered arrangement of experimental materials; Wang, 2017). Conversely, when Gilbert et al. (1988) controlled task difficulty by manipulating error rates, participants assigned to a hard memory task (memorizing a seven-digit number) had higher rejection rates for unfairness than participants assigned to an easy memory condition (memorizing a three-digit number; Gilbert et al., 1988). This apparent inconsistency might be due to the use of different control methods for task difficulty across studies or differences in the behavioral task used to assess fairness consideration. Thus, the findings of these studies should be interpreted with caution.

Although the aforementioned findings are suggestive of an important role of self-contribution and task difficulty in fairness consideration, few studies have manipulated these factors within one experiment. Typically, researchers have analyzed these two factors separately. In real life, both factors are considered and fairness is unlikely to be achieved if individuals consider only relative contribution regardless of difficulty. Thus, the ecological validity of a task or the assessment metric can better be achieved when both factors are considered together, which also would be expected to produce results that are more valuable with respect to solving real-life allocation problems.

The ultimatum game (UG) was adopted in this study to assess the effects of both a contribution factor and a difficulty factor on fairness consideration (Güth et al., 1982; Camerer and Thaler, 1995; Fehr and Fischbacher, 2003). The UG is a widely used paradigm for exploring social decision-making on fairness consideration with key indicators of fairness-related decision-making. In a typical UG, one participant plays the role of a proposer while a second participants acts as the recipient. In each round, the two players are given a certain amount of money (e.g., 50 yuan). The proposer suggests how the money should be distributed, and the recipient accepts or rejects the offer (e.g., 45 yuan to proposer and 5 yuan to recipient). If the recipient accepts the offer, the money is distributed accordingly. If the recipient rejects the offer, nothing is given to either player. Fairness consideration underlies the players choices. In the present study, task difficulty and contribution were manipulated simultaneously in a modified UG task similar to that described in a prior study (Guo et al., 2014).

High temporal resolution event-related potentials (ERPs) can show the time course of brain processing, including processes related to cognition, emotion, and decision-making (Hillyard and Picton, 2011; Luck, 2014). Previous electroencephalogram (EEG) studies investigating the neurophysiological characteristics of fairness consideration in UG decision-making have suggested that unfair offers may induce more negative FRN (feedback-related negativity) amplitudes than fair offers, particularly in recipients with high fairness concerns (Ma et al., 2017; Long et al., 2018; Jin et al., 2020). The FRN is a negative deflection component that appears 200 ~ 350 ms after stimulus onset (Ma et al., 2015; Massi and Luhmann, 2015; Sambrook and Goslin, 2015). FRN amplitude has been related to people’s responses to negative events that violated fairness norms (Boksem and De Cremer, 2010). Source localization analysis has shown that the FRN is derived mainly from the anterior cingulate or medial frontal cortex, a region associated with cognitive control, behavioral decision-making, and conflict monitoring (Gehring and Willoughby, 2002; Polezzi et al., 2010). Some researchers have suggested that the FRN component might reflect conflict between cognition and emotional motivation (Sanfey et al., 2003), especially in response to negative events, such as unfair offers in the UG task (Boksem and De Cremer, 2010; Zhou and Wu, 2011).

The P300 ERP component—which has long been related to higher-order cognitive operations such as selective attention and resource allocation (Polich and Kok, 1995; Hajcak et al., 2010; Massi and Luhmann, 2015; Zheng et al., 2015), especially in response to unexpected stimuli (Olofsson et al., 2008)—has also been related to fairness consideration. The P300 is a late positive peak that occurs 300 ~ 700 ms after the onset of feedback. It has been suggested that the P300 may be sensitive to a top-down outcome evaluation process, in which factors related to the allocation of attentional resources come into play, including reward valence, reward magnitude, and magnitude expectancy (Hajcak et al., 2007; Leng and Zhou, 2010; Zhou and Wu, 2011).

Previous studies implicating self-contribution in fairness consideration have used mostly functional magnetic resonance imaging (Guo et al., 2014). To our knowledge, this study is the first ERP-based fairness consideration study to investigate self-contribution and task difficulty simultaneously. We hypothesized that different degrees of self-contribution and task difficulty would shift individuals’ fairness consideration outcomes. Behaviorally, we predicted that there will be lower acceptance of unfair offers under high self-contribution and hard conditions. Moreover, we predicted that different neural response EEG patterns, including FRN and P300 amplitudes, would be associated with different levels of task difficulty and self-contribution. Specifically, we predicted that FRN amplitude may be more sensitive to fairness concern in a hard condition than in an easy condition whereas P300 amplitude may be more positive in the self-contribution condition than in other-contribution and both-contribution conditions.

## Materials and Methods

### Participants

A sample size calculation performed in G*Power (version 3.1) suggested that 24 participants would ensure 80% statistical power in the case of a medium effect size (effect size of 0.25; alpha level of 0.05) (Faul et al., 2007). We used convenience sampling to recruit research participants through notices posted on Yangzhou University campus. A total of 30 undergraduate and graduate students were enrolled as participants. Three were excluded, including two who reported in the post-experimental questionnaire that they did not believe the setup at all and one whose EEG recording had excessive artifacts. The remaining 27 participants (13 females) included in our statistical analyses had a mean (±standard deviation, SD) age of 21 (±2.4) years.

The participants completed the Chinese version of the Justice Sensitivity Inventory (Schmitt et al., 2010), which contains four subscales (0 = not at all, 5 = very strongly): perpetrator sensitivity, victim sensitivity, beneficiary sensitivity, and observer sensitivity. The participants’ mean scores (±SD) on these subscales were 30.5 (±6.6), 25.6 (±5.5), 27.0 (±6.8), and 23.4 (±3.6), respectively.

All participants were right-handed and had normal or corrected to normal vision. They had no history of any neurological or psychiatric disorders. Written informed consent was obtained from each participant prior to the experiment. All participants were compensated for their participation with a base payment of 30 Chinese yuan and were informed that additional monetary rewards would be paid according to their performance in the task. The base payment was implemented to attract participants and the additional rewards were set to support task completion. In reality, all participants were paid an extra 20 Chinese yuan on top of the base payment.

The experimental procedure adhered to the Declaration of Helsinki and was approved by the Ethical Committee of Yangzhou University. All participants provided informed consent for participating in the study.

### Design and Procedure

The experiment had a 2 × 3 × 2 within-subject design, with the first factor being task difficulty (hard vs. easy), the second being contribution (other-contribution vs. both-contribution vs. self-contribution), and the third being fairness type (fair vs. unfair). Task difficulty was set by manipulating the proportion of correct options in a ball-guessing game. In the easy and hard levels, participants selected which box contains a ball among two boxes (one-in-two) and among four boxes (one-in-four), respectively. Manipulating error rates in this way has been shown previously to alter difficulty (Gilbert et al., 2012). Contribution was set by manipulating hit combinations by participants and partners. Fairness type was defined by offer size with 25:25 being defined as a fair offer and 5:45 and 15:35 being unfair offers.

The experiment was conducted with a 22-inch Lenovo desktop computer as a stimulus presentation device. The computer was equipped with a mouse and a screen with 1,024 × 768 resolution (horizontal and visual angles <5°). The participants were instructed about the rules of the experiment task through an explanation of the written instructions. The experimental preparation, stimulus presentation, and data collection were conducted in E-prime 3 software.

**Figure 1:**
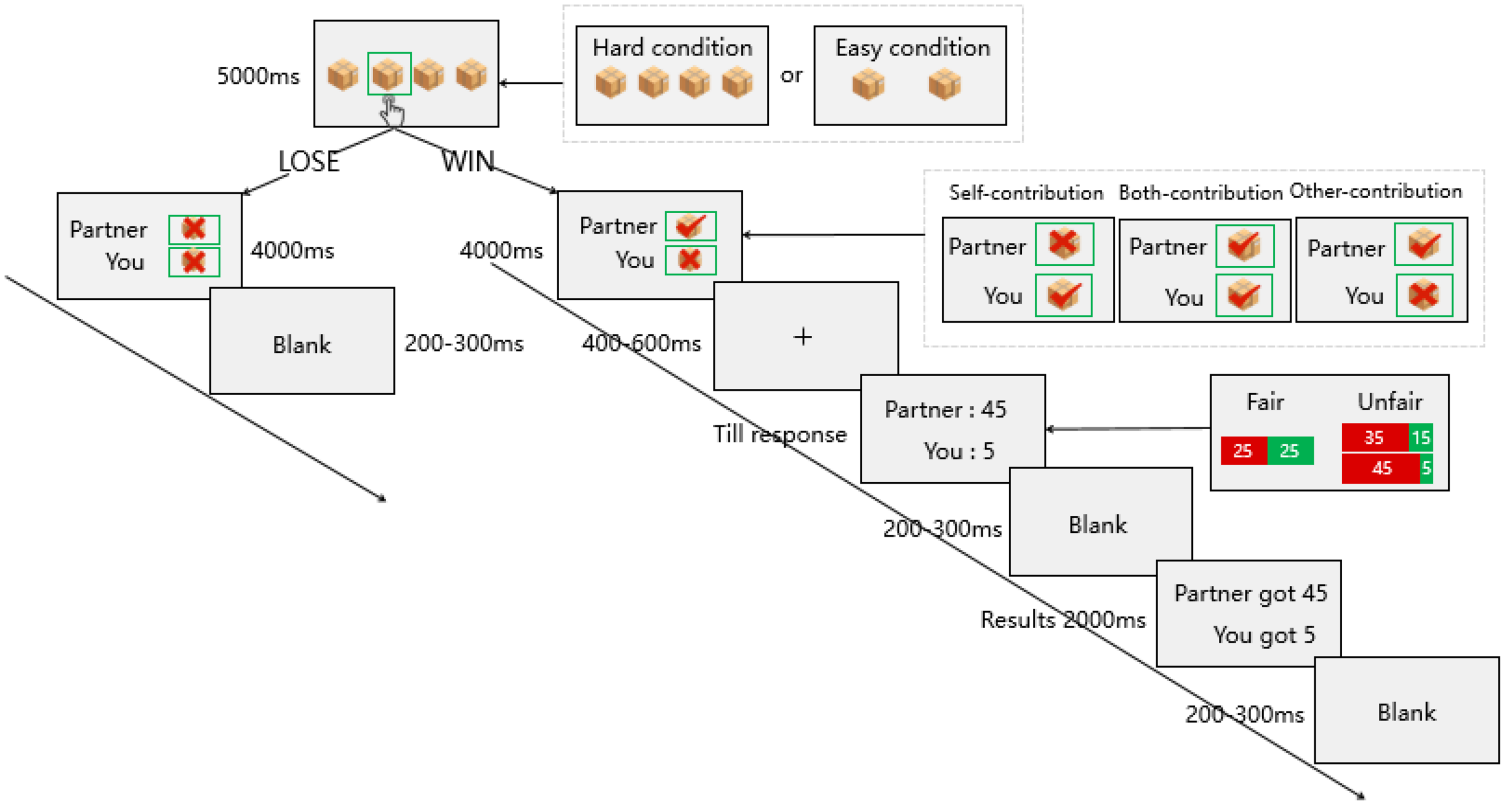
Sequence of events within each trial.

The experimental task consisted of nine 100-trial blocks. At the beginning of each trial, two (easy condition) or four (hard condition) opaque boxes were displayed for 5,000 ms on a computer screen against a grey background, with a small ball hidden in one of the boxes. The participant used their mouse to select the box where the small ball might be, while the partner made an offer decision. Outcomes of participants and partners were presented for 4,000 ms with indications of the respective contribution levels of each participant and their partner. If at least one person in the participant–partner pair guessed the ball location correctly, 50 points were awarded. If neither of them guessed correctly, they received nothing (reward points were converted into payment at the end of experiment). After the presentation of a black fixation point for 400–600 ms, the participant acting in the recipient role would receive a fair or unfair offer (e.g., “Partner: 45, You: 5”) in black Song font (size 32) in the center of the screen. The recipient had to decide to accept or reject each offer. If it was accepted, the recipient and proposer were paid accordingly. Both got nothing if the offer was rejected. Subsequently, the final proposal results were displayed on the screen for 2000 ms. The next trial began after a 200–300 ms blank screen. The trial time course presented in Figure 1.

From the perspective of each participant, the procedure started with meeting one’s “partner” (played by one of our experimental assistants) in the laboratory. To exclude the potential influence of gender and familiarity on fairness consideration, participants were paired with a same-gender assistants (Eckel and Grossman, 1996; Flinkenflogel et al., 2017). Each participant was photographed with their partner standing against the wall with a digital camera. The photographs were used to make the experimental setup more realistic and to ensure that the participants’ believed they were interacting with real offers from real people. Subsequently, the participants in each pair were taken to separate rooms where they worked through a computer network to complete the entire task. The real participants were brought into a dimly lit, electromagnetically shielded room, seated comfortably at a viewing distance of 100 cm from the computer screen. During all trials, participants were instructed to direct their eyes to the center of the computer screen, where a plus sign (+) served as a central fixation point.

To facilitate procedural familiarization, the participants were required to perform 40 practice trials. After the practice trials, the participants entered the formal experiment, in which all proposer’s offers were pre-determined by a computer program and were presented in a pseudo-randomized and balanced.

### EEG Recordings

EEGs were recorded from 64 scalp sites *via* tin electrodes mounted in elastic caps (Neuroscan Inc., Herndon, VA) according to the International 10–20 system. To monitor eye movements and blinks, a vertical electrooculogram (EOG) was recorded with left supraorbital and infraorbital electrodes and a horizontal EOG was recorded by electrodes placed 1.5 cm lateral to the right and left external mastoid. The EEGs and EOGs were amplified by SynAmps2 amplifiers; raw EEG data were referenced online using reference and ground electrodes. All electrode recordings were referenced offline relative to a mean value placed on the left and right mastoid. Continuous sampling was conducted at 1000 Hz/channel for offline analysis with electrode impedances maintained mainly below 5KΩ.

The EEG data were preprocessed with a 0.1–20 Hz (24 Db/oct) band-pass filter and analyzed in MATLAB 2020b with the EEGLAB toolkit. Blink, eye movement, electromyography artifacts were removed from the EEG signals. EEG epochs of 1,000 ms (with a 200 ms pre-stimulus baseline) were extracted offline and time-locked to the onset of each allocation offer. All trials with EEG voltages exceeding a ± 75-μv threshold range during the recording period were excluded from further analysis.

ERPs were identified based on visual inspection and previous ERP waveform studies. The FRN was measured as the peak amplitude in the 120–200-ms time window after the allocation offer onset. The P300 was measured as the peak in the 450–700-ms time window on each electrode. We conducted ERP response analysis on data from electrodes showing the predominant deflections for each ERP: the frontocentral electrode FCz for FRN responses and the parietal electrode Pz for P300 responses.

### Data Analysis

All statistical analyses were conducted in SPSS software (version 22). The behavioral parameters of accepted choice percentage and accepted mean response time were subjected to repeated measures ANOVAs with three within-subjects factors: task difficulty (hard vs. easy), contribution (other-contributed vs. both-contributed vs. self-contributed), and fairness type (fair vs. unfair). If the ANOVAs yielded significant (*p* < 0.05) interactions among task difficulty, self-contribution, and fairness type, then further *F*-tests were conducted to test for simple effects. When significant interactions were observed, interaction analysis was used. The Greenhouse–Geisser correction for violation of the sphericity assumption was applied where appropriate and the Bonferroni correction was used for multiple comparisons.

## Results

### Behavior

**Figure 2:**
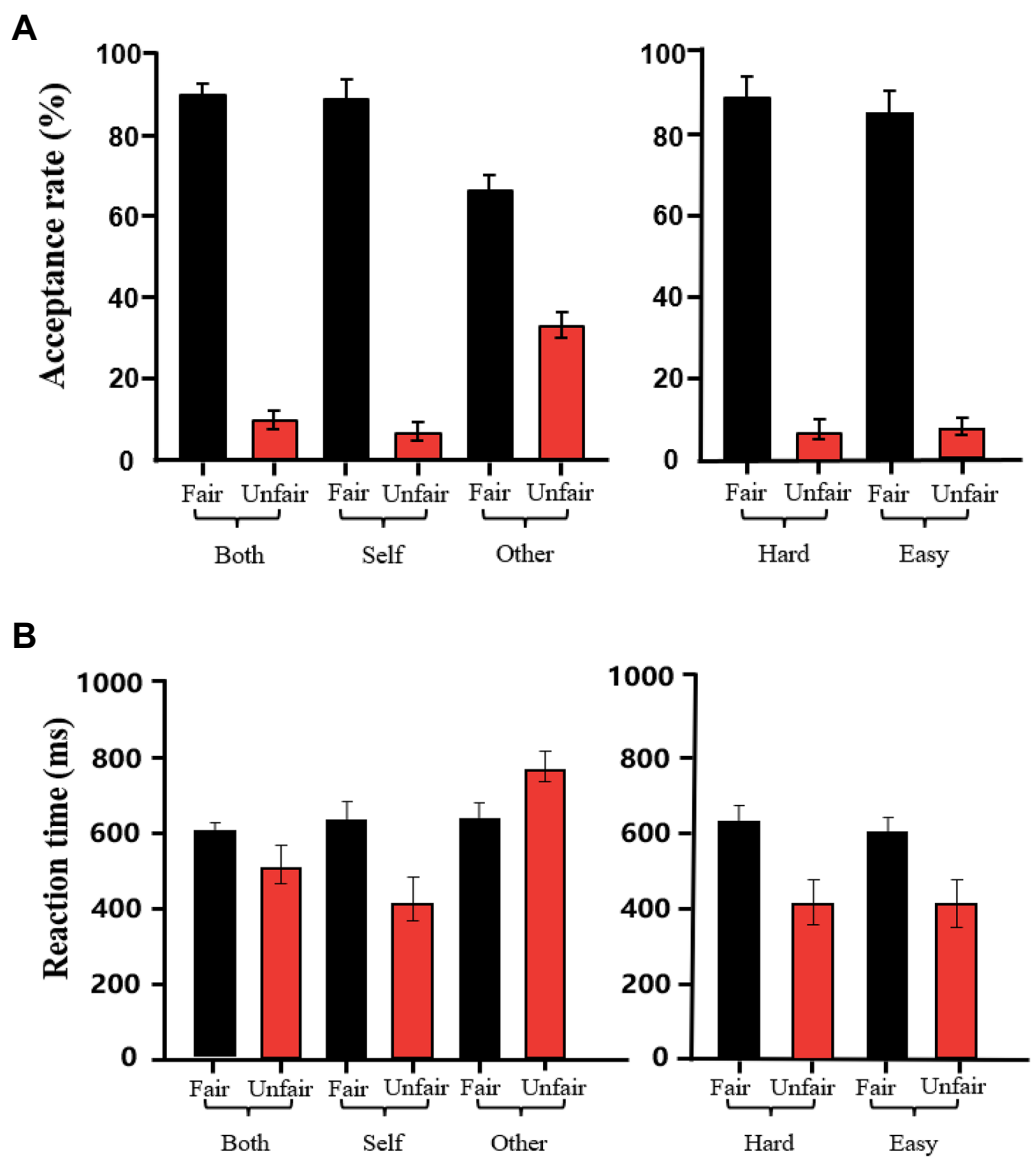
Comparisons of behavioral parameters across conditions. **(A)** Acceptance rates. **(B)** Reaction times. All data are presented as the mean ± SEM.

Acceptance rates for each allocation offer category are reported in Figure 2A. A repeated measures ANOVA revealed a significant main effect of fairness type [*F*(1.26) = 228.03; *p* < 0.01; $\eta_{p}^{2}$ = 0.89], indicating that the acceptance rate for fair offers (81 ± 2.4%) was higher than that for unfair offers (17 ± 2.0%). Although there was no main effect of contribution [*F*(2.52) = 1.85; *p* = 0.16; $\eta_{p}^{2}$ = 0.06], there was a significant interaction between fairness type and contribution [*F*(2.52) = 37.84; *p* < 0.01; $\eta_{p}^{2}$ = 0.59]. The interaction analysis revealed that unfair offers were more acceptable when the recipient was under the other-contribution condition (33 ± 3.2%) than when the recipient was under the self-contribution (7 ± 2.1%) or both-contribution (10 ± 2.2%) condition (*p*s < 0.01). In the fair condition, acceptance rates for fair offers did not differ significantly between the both- and self-contribution conditions (*p* = 0.21) and there was not a significant main effect of task difficulty [*F*(1.26) *=* 1.02; *p* = 0.32; $\eta_{p}^{2}$ = 0.03]. The other two-way interactions [task difficulty × fairness type: *F*(1.26) = 3.09; *p* = 0.09; $\eta_{p}^{2}$ = 0.10; contribution × task difficulty: *F*(2.52) = 1.02; *p* = 0.36; $\eta_{p}^{2}$ = 0.03] and the three-way interaction [task difficulty × contribution × fairness type: *F*(2.52) = 0.32; *p* = 0.72; $\eta_{p}^{2}$ = 0.01] failed to reach statistical significance.

Mean reaction times for each allocation offer category are shown in Figure 2B. A repeated measures ANOVA revealed a significant main effect of contribution [*F*(2.52) = 18.39; *p* < 0.01; $\eta_{p}^{2}$ = 0.28]. Post-hoc analysis showed that the recipients exhibited faster mean reaction times in the self-contribution condition (527 ± 33 ms) than in the other-contribution (720 ± 32 ms; *p* = 0.01) or both-contribution (567 ± 21 ms; *p* < 0.01) conditions. Reaction times for the both- and other-contribution conditions were similar (*p* = 0.54). There was also a significant main effect of fairness type [*F*(1.26) = 6.18; *p* = 0.04; $\eta_{p}^{2}$ = 0.12], with recipients exhibiting a faster reaction time in the unfair condition (572 ± 27 ms) than in the fair condition (638 ± 23 ms). There was a significant interaction between fairness type and contribution [*F*(2.52) =18.57; *p* < 0.01; $\eta_{p}^{2}$ = 0.30]. The interaction analysis revealed that the reaction time difference between fair and unfair offers was greater in the self-contribution condition (215 ± 11 ms) than in the other-contribution (137 ± 5 ms) and both-contribution (101 ± 23 ms) conditions (*p*s < 0.05). However, no significant main effect of task difficulty was found [*F*(1.26) = 2.10; *p* = 0.16; $\eta_{p}^{2}$ = 0.07]. No interaction was found between the contribution and task difficulty [*F*(2.52) = 0.88; *p* = 0.42; $\eta_{p}^{2}$ = 0.03], nor between task difficulty and fairness type [*F*(1.26) = 0.74; *p* = 0.39; $\eta_{p}^{2}$ = 0.02]. No significant three-way interaction was found among task difficulty, contribution, and fairness type [*F*(2.52) = 0.45; *p* = 0.63; $\eta_{p}^{2}$ = 0.04].

We also allocated participants into high-score and low-score groups based on their scores on the justice sensitivity inventory. There were no significant differences between these two justice sensitivity groups on UG task (*t* = 0.68, *p* = 0.50), with 91.99 ± 10.8% acceptance rate of fair offers in high-score group, and 88.57 ± 12.87% in low-score group, demonstrating that our results should not be sensitive to justice sensitivity.

### Event-Related Potentials

#### The FRN

**Figure 3:**
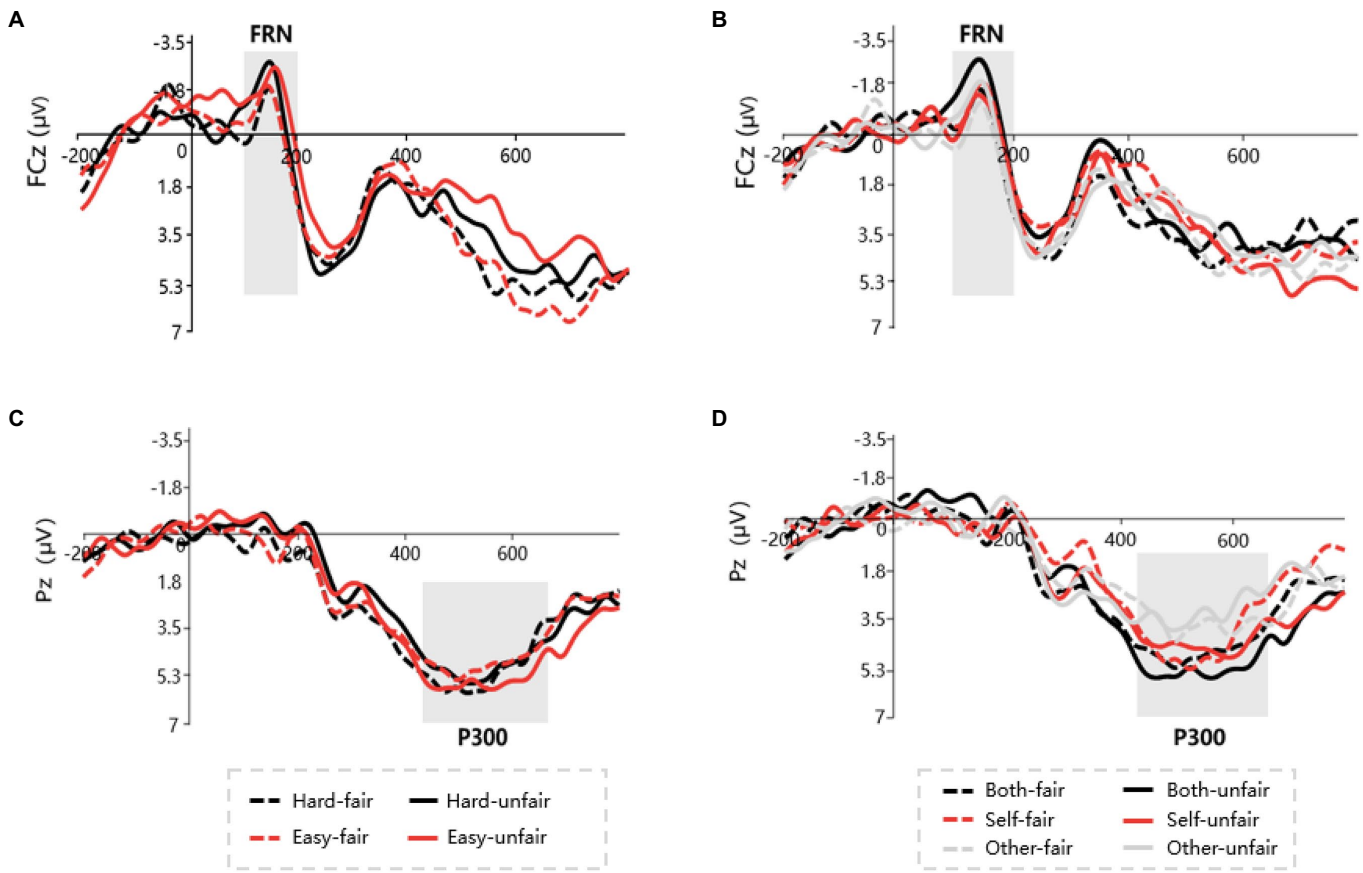
ERP grand average waveforms. **(A)** FRN waveforms across difficulty and fairness conditions. **(B)** FRN waveforms across contribution and fairness conditions. **(C)** P300 waveforms across difficulty and fairness conditions. **(D)** P300 waveforms across contribution and fairness conditions.

**Figure 4:**
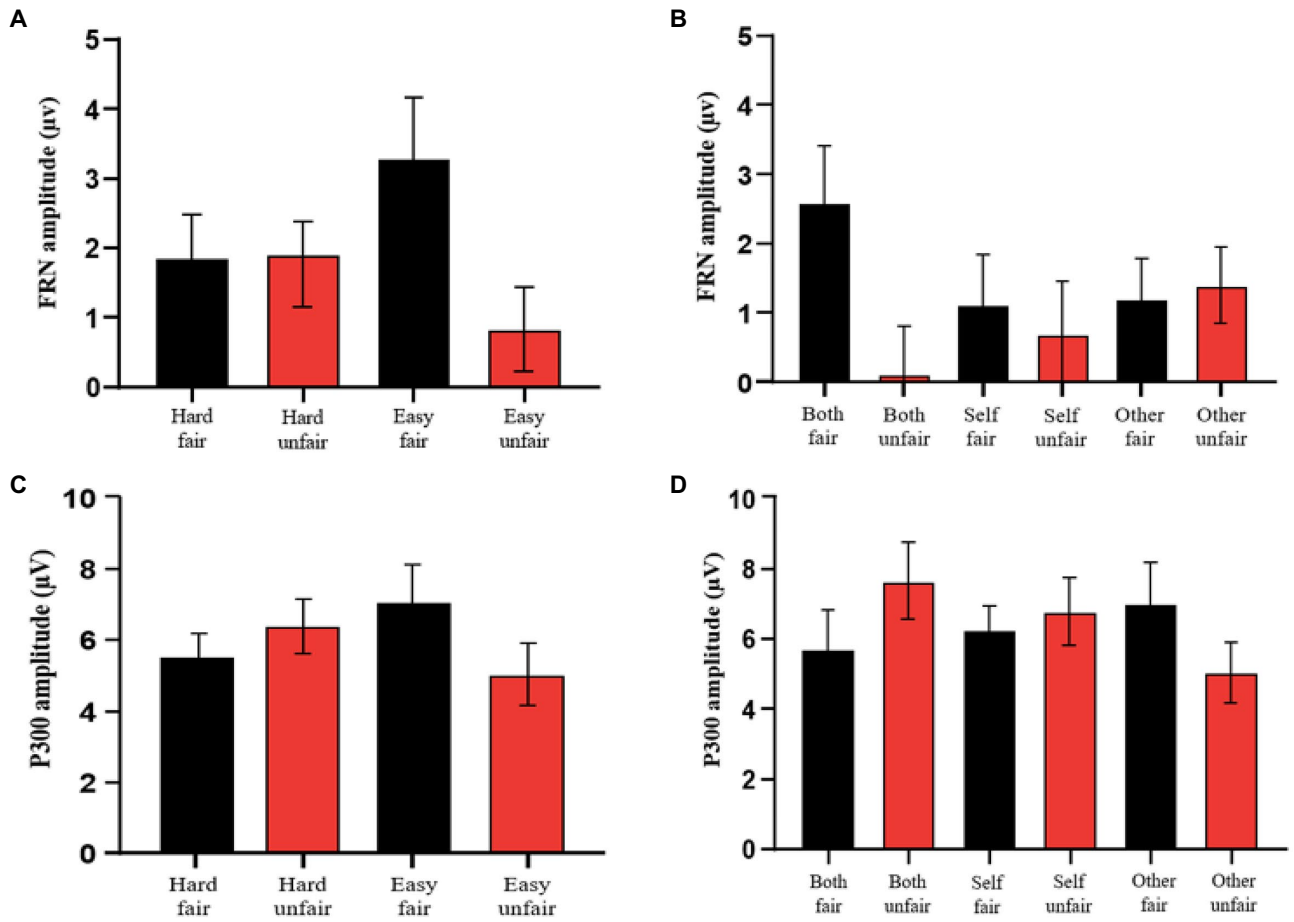
ERP amplitudes. **(A)** Comparison of FRN amplitudes across difficulty and fairness conditions. **(B)** Comparison of FRN amplitudes across contribution and fairness conditions. **(C)** Comparison of P300 amplitudes across difficulty and fairness conditions. **(D)** Comparison of P300 amplitudes across contribution and fairness conditions.

At least 50 averaged EEGs were obtained in each condition for each of the 27 participants. Grand waveforms obtained with 0.1–20-Hz band-pass filtering are shown for each experimental condition in Figures 3A,B, and the FRN amplitudes are shown in Figures 4A,B. There were not significant main effects of task difficulty [*F*(1.26) = 0.28; *p* = 0.59; $\eta_{p}^{2}$ = 0.11], contribution [*F*(2.52) = 0.56; *p* = 0.57; $\eta_{p}^{2}$ = 0.02] or fairness type [*F*(1.26) = 8.61; *p* = 0.24; $\eta_{p}^{2}$ = 0.07]. There was a significant interaction between task difficulty and fairness type [*F*(1.26) = 6.52; *p* = 0.003; *η_p_^2^* = 0.29], but no other significant interactions [task difficulty × contribution *F*(2.52) = 1.81, *p* = 0.17, $\eta_{p}^{2}$ *=* 0.06; contribution × fairness type *F*(2.52) = 2.11, *p* = 0.13, $\eta_{p}^{2}$ = 0.07; fairness type × task difficulty × contribution *F*(2.52) = 5.98, *p* = 0.18, $\eta_{p}^{2}$ = 0.05]. The interaction analysis revealed that the FRN effect (unfair minus fair) differed between the hard condition (0.20 ± 0.03 μv) and the easy condition (−2.31 ± 0.38 μv), t(26) = 0.95, *p* = 0.03.

#### The P300

Grand mean waveforms obtained for each experimental condition after 20-Hz low-pass filtering are shown in Figures 3C,D, and the P300 amplitudes are shown in Figures 4C,D. A repeated measures ANOVA of P300 amplitude (central posterior electrode) with task difficulty, contribution, and fairness type as within-subjects variables revealed a significant interaction between contribution and fairness type [*F*(2.52) = 10.85; *p* < 0.001; $\eta_{p}^{2}$ = 0.294]. The interaction analysis revealed that the P300 effect (unfair minus fair) was larger in the both-contribution condition (1.95 ± 0.03 μv) than in the self-contribution condition (0.52 ± 0.26 μv) or other-contribution condition (−1.98 ± 0.27 μv; *p*s < 0.01). There were no other significant main effects [task difficulty: *F*(1.26) = 2.75; *p* = 0.10; $\eta_{p}^{2}$ = 0.09; contribution: *F*(2.52) = 0.62; *p* = 0.54; $\eta_{p}^{2}$ = 0.02; fairness type: *F*(1.26) = 0.19; *p* = 0.66; $\eta_{p}^{2}$ = 0.007] or interactions [task difficulty × contribution *F*(2.52) = 1.21; *p* = 0.35; $\eta_{p}^{2}$ = 0.03; task difficulty × fairness type *F*(1.26) = 0.27; *p* = 0.60; $\eta_{p}^{2}$ = 0.01; task difficulty × contribution × fairness type *F*(2.52) = 4.57; *p* = 0.15; $\eta_{p}^{2}$= 0.05].

## Discussion

In the present study, we investigated whether one’s own contribution and task difficulty moderate fairness consideration behaviorally or influence neurophysiological responses during performance of the UG. Behaviorally, the participants were more likely to reject unfair offers in the self-contribution condition than in the both- and other-contribution conditions. Neurophysiologically, unfair offers allocated in hard condition trials induced more negative-going FRN responses in the early time window than similar offers in easy condition trials. More positive P300 potentials were observed in the both-contribution condition than in the self- and other-contribution conditions.

Our behavioral results are similar to classic UG findings in that people tended to reject unfair offers and are further consistent with Guo et al.’s (2014) findings in indicating that self-contribution make participants less likely to accept unfair offers than when they are in other-contribution or both-contribution trials (Guo et al., 2014). Hence, it appears that the participants tended to accept offers when they were in line with their own contribution proportions. Otherwise, they would often punish the proposer, apparently as a form of retaliation and defiance, by refusing unfair offers in agreement with the pattern of the distributive justice theory proposed by Hoffman and Spitzer (Hoffman and Spitzer, 1985). Unfair treatment by a low-contributing partner may induce negative emotions that would reinforce the participants’ willingness to enact punishment, resulting in punitive decisions and thus rejection of unfair offers.

Interestingly, although we did not find behavioral effects of task difficulty on fairness decisions, we did see effects of task difficulty on our EEG results. This disassociation may be due to neurophysiological responses were sensitive to difficulty level. Another significant issue is that we did not observe a significant interaction of task difficulty and contribution in our experiment. There are a number of potential, attemptable methods of setting task difficulty that might better differentiate levels of difficulty (Gilbert et al., 2012). It might be that the complexity of the experimental design in terms of multiple conditions masked difficulty effects to some extent.

Importantly, our results revealed that unfair offers induced larger FRNs in hard condition trials than in easy condition trials. It has been suggested that FRN amplitudes may be heavily dependent on how concerned subjects are about the decision outcome, especially in the context of social interactions (Luo et al., 2011). In other words, the FRN may reflect the magnitude of outcome value. Indeed, previously, unfair offers have been reported to result in significantly larger FRN amplitudes than fair ones, especially in individuals with a high-level of concern for fairness norms (Boksem and De Cremer, 2010). In fact, FRN components originating from the anterior cingulate cortex have previously been associated with conflict monitoring (Botvinick et al., 1999; MacDonald et al., 2000). Some researchers have argued that this conflict may reflect a contradiction between the desire to accept monetary benefits and an aversion to unfair treatment (Van Lange et al., 2013). In our experiment, when participants encountered unfair offers in easy condition trials, the expression of aversive emotions may have needed to be somewhat inhibited to enable them to accept an unfair benefit allocation. However, such conflicts would be suppressed in the hard condition in order to protect one’s personal interests.

The present finding of a more positive P300 response in the both-contribution condition than in the self- and other-contribution conditions was unexpected. We speculate that this result might be attributed to participants’ level of involvement in the cooperative process such that when the two contributions were balanced, both partners showed a well-coordinated and equivalent performance. Such dynamic engagement is essential to maintaining partner cooperation. Alternatively, this surprising result could be related to our use of different evaluation criteria relative to prior studies. Previously, P300 has been reported to be associated with higher-order cognitive operations, such as attentional resource allocation and motivational/affective evaluation (Gray et al., 2004; Linden, 2005). Participants exhibiting more positive choices in the both-contribution condition might be driven by external motivations, such as maintaining a good image in front of the researcher. Choices under the other two contributions were more likely to be motivated by self-interest. Alternatively, P300 differences may reflect differences in top-down outcome evaluation processes (Yeung and Sanfey, 2004; Sato et al., 2005). Stronger P300 responses in the both-contribution condition, than in the self- and other-contribution conditions, may indicate that more attentional resources were invested in sustaining relationships in the both-contribution condition.

Despite the significant results obtained in the present study, there are some limitations. Firstly, we did not observe significant effects of task difficulty on behavioral responses, indicating that the task difficulty shift lacked salience. Secondly, we examined fairness consideration with a single task, UG, which has been a popular paradigm for characterizing fairness-related decision-making. Future research could incorporate more paradigms (e.g., dictator games or impunity games) to further explore fairness consideration. Thirdly, the fact that only healthy university students were included as subjects may limit the representativeness of the present results. Future studies should consider including a greater diversity of participants, including people of different ages (e.g., elderly and children) and neurology patients (e.g., brain injury patients). Finally, because this study was conducted in the context of a highly collectivist culture, the results might not be generalizable to participants from other cultural backgrounds. In future research, accounting for this variable may yield more accurate and in-depth findings regarding how factors affect fairness consideration.

## Conclusion

The present findings underscore the influence of self-contribution on fairness consideration and demonstrate neurophysiological responses during fairness consideration over the FRN and P300 time phases. Participants were inclined to make more altruistic choices when their contributions to the reward were relatively small compared to those of the proposer. As expected, task difficulty influenced neurophysiological responses during offer evaluation in addition to other criteria in fairness consideration. Unfair offers in a hard task may be judged as more violative of expectations than in an easy condition, thereby inducing more pronounced FRN amplitudes early in the stimulus processing period. At a later stage, larger P300 amplitudes were observed in both-contribution condition trials than in self-contribution or other-contribution condition trials. Our findings provide new evidence for understanding and explaining the effects of task difficulty and self-contribution on fairness evaluation in cooperative contexts.

## Data Availability Statement

The raw data supporting the conclusions of this article will be made available by the authors, without undue reservation.

## Ethics Statement

The study was reviewed and approved by the Ethics Committee of Yangzhou University. The participants provided their written informed consent to participate in this study.

## Author Contributions

WG and BW conceived and designed the experiments. LX and BW performed the experiments and analyzed the data. LX and WG wrote the manuscript. All authors contributed to the article and approved the submitted version.

## Funding

This work was supported by the National Social Science Foundation of China (Grant No. 18CTY014).

## Conflict of Interest

The authors declare that the research was conducted in the absence of any commercial or financial relationships that could be construed as a potential conflict of interest.

## Publisher’s Note

All claims expressed in this article are solely those of the authors and do not necessarily represent those of their affiliated organizations, or those of the publisher, the editors and the reviewers. Any product that may be evaluated in this article, or claim that may be made by its manufacturer, is not guaranteed or endorsed by the publisher.
